# Supplementary material for: Probiotic Ingestion, Obesity, and Metabolic-Related Disorders: Results from NHANES, 1999–2014
Source: Nutrients. 2019 Jun 28;11(7):1482. doi: 10.3390/nu11071482 (PMC6683043; doi:10.3390/nu11071482)
Supplement: Supplementary file 1 [file nutrients-11-01482-s001.pdf]

Supplementary data

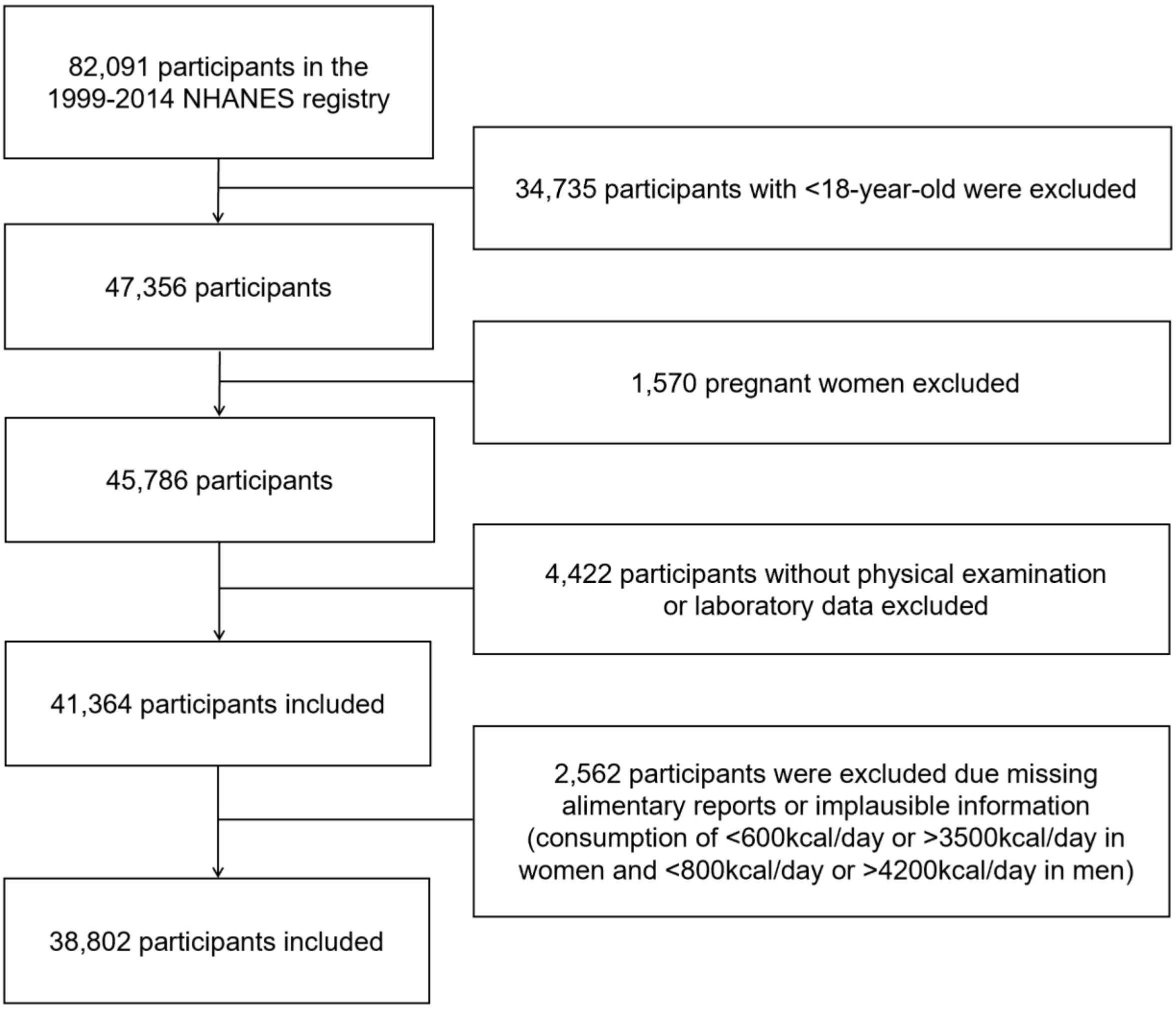

Figure S1. - Flowchart of the study population

Table S1. – Probiotic supplements included.

21ST CENTURY HIGH POTENCY ACIDOPHILUS
365 PROBIOTIC COMPLEX WITH ACIDOPHILUS DOUBLE STRENGTH
4X PROBIOTIC UP&UP CONTAINS 4 STRAINS OF NATURAL, BENEFICIAL BACTERIA WITH B. INFANTIS 10 MG
ACCUFLORA ADVANCED CD PROBIOTIC 5 PROBIOTIC STRAINS ADVANCED CD: CONTROLLED DELIVERY
ADVANCED NATURALS FLORAMAX ADVANCED PROBIOTIC FORMULA 12 BILLION CELLS OF 7 STRAINS OF MICROFLORA WITH ACIDOPHILUS, BIFI
ALIGN PROBIOTIC
ALIGN PROBIOTIC B. INFANTIS 35624 WITH: UNIQUE B. INFANTIS 35624
ALLERGY RESEARCH GROUP BIFIDOBIOTICS WITH L. SPOROGENES
ALLERGY RESEARCH GROUP GI FLORA L.ACIDOPHILUS, L.CASEI, L. RHAMNOSUS, B. LONGUM HYPOALLERGENIC
AMERICAN HEALTH CHEWABLE ACIDOPHILUS WITH BIFIDUS MILK FREE
AMERICAN HEALTH CHEWABLE MILK FREE ACIDOPHILUS AND BIFIDUM ONE BILLION ORGANISMS VEGETARIAN FORMULA
BACID PROBIOTIC CONTAINS A BLEND OF BENEFICIAL BACTERIA INCLUDING LACTOBACILLUS ACIDOPHILUS
BASIC'S NATURAL ACIDOPHILUS BASIC VITAMINS
BIOGAIA PROBIOTICS GERBER SOOTHE COLIC DROPS PROBIOTIC
BIORAY CYTOFLORA PROBIOTIC IMMUNITY TONIC DAILY
BIOSPEC PROBIOTIC PLUS HIGH POTENCY, MULTI-STRAIN PROBIOTIC
BLUEBONNET MILK-FREE ACIDOPHILUS PLUS FOS L. ACIDOPHILUS, L. BULGARICUS, BIFIDUS
BREAKTHROUGH FORMULATIONS MEGA-FLORA DIGESTIVE TRACT FLORA
CELL NUTRITIONALS PROBIOTICS AR ACID RESISTENT 16 STRAIN FORMULA COMPREHENSIVE, DAIRY FREE FORMULA PROVIDING 12 BILLION VIABLE CELLS PER CAPSULE AT TIME OF MANUFACTURE
COUNTRY LIFE DAIRY-FREE ACIDOPHILUS WITH PECTIN
COUNTRY LIFE POWER-DOPHILUS WITH FOS HYPOALLERGENIC MILK FREE
CULTURELLE DIGESTIVE HEALTH PROBIOTIC PROBIOTIC WITH NATURALLY SOURCED DAIRY-FREE LACTOBACILLUS GG ONCE DAILY CAPSULES 10 BILLION ACTIVE CULTURES
CULTURELLE KIDS! PACKETS PROBIOTIC PROBIOTIC WITH NATURALLY SOURCED LACTOBACILLUS GG
CULTURELLE WITH LACTOBACILLUS GG
CVS PHARMACY EXTRA STRENGTH ACIDOPHILUS
DDS-100 ACIDOPHILUS WITH FOS CHEWABLE TABLETS (2 BILLION CFU/G) UAS LABORATORIES
DEFAULT LACTOBACILLUS ACIDOPHILUS
DEFAULT PROBIOTIC
DESIGNS FOR HEALTH PROBIOTIC SYNERGY POWDER 20 BILLION ORGANISMS PER SERVING

DIGESTIVE ADVANTAGE LACTOSE DEFENSE FORMULA DUAL-ACTION! ENZYME PLUS PROBIOTICS TAKE EVERY DAY
DIGESTIVE ENZYMES & PROBIOTICS WITH ENZYGUARD-D 4LIFE
DIGESTZEN PB ASSIST+ PROBIOTIC DEFENSE FORMULA DOTERRA
ECOQUEST / INFINITY ESSENTIALS FOR LIFE 2 ENZYME, FLORA AND CHROMIUM COMPLEX
ENERGETIX FLORA SYNERGY
ENZYMATIC THERAPY NATURAL MEDICINES ACIDOPHILUS PEARLS WITH L. ACIDOPHILUS AND B. LONGUM
ENZYMATIC THERAPY PROBIOTIC PEARLS HIGH POTENCY ADVANCED 3-LAYER SOFTGEL ONCE DAILY LACTOBACILLI & BIFIDOBACTERIA
FINEST NUTRITION ACIDOPHILUS L. ACIDOPHILUS 1 BILLION CFUS (LIVE ORGANISMS) PER TABLET ONE PER DAY
FLORA BILBERRY EXTRACT 25% STANDARDIZED ANTHOCYANIDINS WITH FREEZE-DRIED BLUEBERRY POWDER
FLORA CERTIFIED ORGANIC FLAX OIL COLD-PRESSED & UNREFINED
FLORA FLOR-ESSENCE
FLORA UDO'S CHOICE OIL BLEND CERTIFIED ORGANIC BLEND OF FLAX AND OTHER NUTRITIONALLY SUPERIOR OILS
FLORA UDO'S CHOICE UDO'S OIL DHA 3-6-9 BLEND BASED ON THE IDEAL 2:1:1 RATIO OF OMEGA FATTY ACIDS
FLORA WHITE WILLOW BARK 55 MG STANDARDIZED SALICIN
FLORABABY ADVANCED PROBIOTIC FORMULA FOR INFANTS & TODDLERS 5 STRAINS OF BENEFICIAL PROBIOTICS 4 BILLION LIVE CULTURES PER SERVING PROBIOTIC RENEW LIFE
FLORADIX CALCIUM-MAGNESIUM WITH ZINC, VITAMIN D AND HERBS
FLORADIX FLORAVITAL IRON + HERBS LIQUID EXTRACT FORMULA RICH IN IRON AND B-VITAMINS VEGETARIAN LIQUID FORMULA
FLORADIX IRON + HERB TABLETS HIGH POTENCY IRON WITH VITAMINS, YEAST AND HERB EXTRACTS
FLORADIX KINDER LOVE CHILDREN'S MULTIVITAMIN LIQUID EXTRACT FORMULA VITAMINS A, B, C, D AND E WITH CALCIUM, HERBAL EXTRA
FLORADIX TABLETS WITH IRON, VITAMINS, YEAST AND HERB EXTRACTS
FLORAJEN3 HIGH POTENCY PROBIOTIC ACIDOPHILUS BIFIDUM LONGUM 15 BILLION LIVE CULTURES PER CAPSULE
FRIENDLY FLORA PREBIOTIC AND PROBIOTIC COMPLEX FOS AND LACTOBACILLUS 5 BILLION CFU PROCAPS LABORATORIES
FUSION PLUS IRON / FOLIC ACID / VITAMIN / PROBIOTIC CAPSULES
FUTUREBIOTICS COLON GREEN GENTLE, ALL-NATURAL FIBER WITH PROBIOTICS AND ENZYMES NOW WITH SUPER-STABLE PROBIOTICS
GARDEN OF LIFE PRIMAL DEFENSE HSO PROBIOTIC FORMULA
GARDEN OF LIFE PRIMAL DEFENSE ULTRA ULTIMATE PROBIOTIC FORMULA 15 BILLION CELLS DAILY 13 BENEFICIAL CULTURES WITH HSOS W

GARDEN OF LIFE RAW BEYOND VITAMINS & MINERALS VITAMIN CODE 50 & WISER MEN RAW WHOLE FOOD MULTI LIVE PROBIOTICS & ENZYMES RAW FOOD-CREATED NUTRIENTS WITH CODE FACTORS RAW FOOD
GARDEN OF LIFE RAW PROBIOTICS WOMEN 50 & WISER 85 BILLION LIVE CULTURES 33 PROBIOTIC STRAINS PROBIOTIC-CREATED VITAMINS, MINERALS, ENZYMES & PREBIOTICS CONTAINS TARGETED PROBIOTICS RAW WHOLE FOOD
GARDEN OF LIFE RAW VITAMIN CODE 50 & WISER WOMEN RAW WHOLE FOOD MULTI LIVE PROBIOTICS & ENZYMES RAW FOOD-CREATED NUTRIEN
GENERIC20 GR8-DOPHILUS 8 STRAINS & 4 BILLION POTENCY WITH FOS
GENERIC36 ACIDOPHILUS PROBIOTIC BLEND
GENERIC7 PROBIOTIC ACIDOPHILUS
GENERIC8 ACIDOPHILUS & PROBIOTIC COMPLEX
GNC NATURAL BRAND ACIDOPHILUS 2 BILLION CFU
GNC NATURAL BRAND ACIDOPHILUS 4 BILLION CFU
GNC NATURAL BRAND MEGA ACIDOPHILUS
GNC NATURAL BRAND POTENT ACIDOPHILUS
GNC NATURAL BRAND ULTRA ACIDOPHILUS 350 MG
GNC PROBIOTIC COMPLEX CHEWABLE TABLET 1.5 BILLION CFUS
GNC PROBIOTICS PROBIOTIC COMPLEX 4 4 BILLION CFUS
GNC PROBIOTICS ULTRA 25 BILLION CFUS PROBIOTIC COMPLEX
GNC ULTRA 50 BILLION CFUS PROBIOTIC COMPLEX GUARANTEED POTENCY 50
GNC WOMEN'S ULTRA MEGA WITH PROBIOTICS MULTIVITAMIN DELIVERS 1 BILLION LIVE, ACTIVE CULTURES WITH 1,600 IU OF VITAMIN D-3
HEALTH PLUS INC. SUPER COLON CLEANSE WITH HERBS & ACIDOPHILUS
HEALTHWAY MEDICAL, P.C. PRO-C 750 MG VITAMIN C WITH PROBIOTICS VEGETARIAN
HEALTHY ORIGINS NATURAL PROBIOTIC 30 BILLION CFU'S 8 STRAINS AND 30 BILLION COLONY FORMING UNITS FLORAFIT
HERBALIFE FLORAFIBER LACTOBACILLUS ACIDOPHILUS AND FIBER
JARROW FORMULAS BABY JARRO-DOPHILUS +FOS WITH CLINICALLY DOCUMENTED STRAINS 3 BILLION PROBIOTIC
JARROW FORMULAS BABY'S JARRO-DOPHILUS WITH CLINICALLY DOCUMENTED STRAINS 3 BILLION PER GRAM HYPOALLERGENIC NON-DAIRY PRO
JARROW FORMULAS ENHANCED PROBIOTIC SYSTEM JARRO-DOPHILUS EPS ENTERIC COATED
JARROW FORMULAS JARRO-DOPHILUS + FOS 6 BENEFICIAL PROBIOTIC STRAINS WITH CLINICALLY DOCUMENTED STRAINS 3.4 BILLION PER CAPSULE PROBIOTIC
JARROW FORMULAS JARRO-DOPHILUS ORIGINAL WITH CLINICALLY DOCUMENTED STRAINS 6 BENEFICIAL PROBIOTIC STRAINS 3.4 BILLION PER CAPSULE PROBIOTIC
JUICE FOR LIFE POWER FRUIT FORMULA WHOLE FOOD CONCENTRATES WITH ENZYMES, PROBIOTICS & ANTIOXIDANTS
JUICEFESTIV FRUITFESTIV 23 FRUITS, ANTIOXIDANTS, PROBIOTICS & DIGESTIVE ENZYMES FEATURES: ACAI, POMEGRANATE, BLUEBERRY AND NONI NATROL

KID'S KYO-DOPHILUS PROBIOTIC ONE PER DAY
KLAIRE LABS THER-BIOTIC INFANT FORMULA 10+ BILLION CFUS MULTI-SPECIES PROBIOTIC
KYO-DOPHILUS
KYO-DOPHILUS PROBIOTICS PLUS CRANBERRY EXTRACT
L'IL CRITTERS PROBIOTIC ACIDOPHILUS DAILY
L.A. FARMACIA NATURAL MICROENCAPSULATED ACIDOPHILUS
LIFETIME LIQUID ACIDOPHILUS SOY BASE MILK FREE
MASON NATURAL CHEWABLE SOLUBLE FIBER WITH PROBIOTICS ADULTS & KIDS
MEGAFLORA OPTIMAL POTENCY PROBIOTIC FORMULA 100% WHOLE FOOD MEGAFOOD
METAGENICS LACTO VIDEN ID DAIRY FREE, STRAIN MODIFIED LACTOBACILLUS BLEND
METAGENICS ULTRA FLORA PLUS DF CAPSULES
METAGENICS ULTRAFLORA IMMUNE HEALTH PROBIOTIC
MILK, COW'S, FLUID, ACIDOPHILUS, 1% FAT
MILK, COW'S, FLUID, ACIDOPHILUS, 2% FAT
NATREN'S MEGA DOPHILUS DAIRY CAPSULES
NATREN'S MEGA DOPHILUS DAIRY POWDER
NATROL ACIDOPHILUS PROBIOTIC
NATURAL FACTORS MULTI ACIDOPHILUS WITH FOS CAPSULES 4 BILLION ACTIVE CELLS
NATURE CITY TRUELIFE PB PROBIOTIC AND PREBIOTIC BLEND PROFESSIONAL STRENGTH! 30 BILLION LIVE 'GOOD BACTERIA' CELLS PER DOSE PROVIDES 6 HEALTH BOOSTING PROBIOTIC STRAINS
NATURE MADE ACIDOPHILUS 500 MILLION LIVE CELLS PER TABLET
NATURE MADE ACIDOPHILUS PROBIOTICS 1 BILLION LIVE CELLS PER SERVING
NATURE MADE TRIPLE PROBIOTIC
NATURE'S BLEND HIGH POTENCY CHEWABLE ACIDOPHILUS
NATURE'S BLEND PROBIOTIC BLEND
NATURE'S BOUNTY ACIDOPHILUS
NATURE'S BOUNTY ACIDOPHILUS PROBIOTIC 100 MILLION ORGANISMS LACTOBACILLUS ACIDOPHILUS 1 PER DAY TABLET
NATURE'S BOUNTY CHEWABLE PROBIOTIC ACIDOPHILUS 1 BILLION ORGANISMS WITH BIFIDUM CHEWABLE MILK FREE WAFER
NATURE'S BOUNTY PROBIOTIC ACIDOPHILUS
NATURE'S BOUNTY PROBIOTIC ACIDOPHILUS 100 MILLION ORGANISMS LACTOBACILLUS ACIDOPHILUS 1 PER DAY CAPSULE
NATURE'S BOUNTY PROBIOTIC ACIDOPHILUS CAPSULES
NATURE'S BOUNTY ULTRA STRENGTH ADVANCED PROBIOTIC 10 10 PROBIOTIC ORGANISMS WITH ACTIVE CULTURE SUPPORT 20 BILLION LIVE PROBIOTIC CULTURES
NATURE'S LIFE LACTOBACILLUS ACIDOPHILUS APPLE PECTIN LIVE, ACTIVE ORGANISMS PROBIOTIC CAPSULES
NATURE'S PLUS ADULT'S EAR, NOSE & THROAT LOZENGES WITH K12 PROBIOTICS

NATURE'S PLUS SOURCE OF LIFE ANIMAL PARADE ACIDOPHIKIDZ WITH PROBIOTICS, FOS & RHODODENDRON CHILDREN'S CHEWABLE WITH WHO
NATURE'S SUNSHINE BIFIDOPHILUS FLORA FORCE BIFIDOBACTERIUM LONGUM & LACTOBACILLUS ACIDOPHILUS 470 MG
NATURE'S SUNSHINE BIFIDOPHILUS FLORA FORCE PROBIOTIC 4 BILLION TOTAL MICROORGANISMS PER CAPSULE
NATURE'S SUNSHINE PROBIOTIC ELEVEN
NATURE'S WAY ONCE DAILY PRIMADOPHILUS
NATURE'S WAY ONCE DAILY PRIMADOPHILUS CHILDREN POWDER WITH FOS TRUE IDENTITY BIFIDOBACTERIA & LACTOBACILLI TRUE POTENCY
NATURE'S WAY ONCE DAILY PRIMADOPHILUS CHILDREN POWDER WITH SCFOS TRUE IDENTITY BIFIDOBACTERIA & LACTOBACILLI TRUE POTENCY 3 BILLION CFU
NATURE'S WAY ONCE DAILY PRIMADOPHILUS FOR CHILDREN
NATURE'S WAY ONCE DAILY PRIMADOPHILUS JUNIOR TRUE IDENTITY LACTOBACILLI & BIFIDOBACTERIA TRUE POTENCY 3 BILLION CFU TRUE
NATURE'S WAY ONCE DAILY PRIMADOPHILUS KIDS TRUE POTENCY 3 BILLION CFU TRUE IDENTITY LACTOBACILLI & BIFIDOBACTERIA ORANGE
NATURE'S WAY ONCE DAILY PRIMADOPHILUS KIDS TRUE POTENCY 3 BILLION CFU TRUE IDENTITY LACTOBACILLI & BIFIDOBACTERIA ORANGE AGES 2-12
NATURE'S WAY PRIMADOPHILUS BIFIDUS ONCE DAILY TRUE POTENCY 5 BILLION CFU TRUE IDENTITY BIFIDOBACTERIA & LACTOBACILLI TRU
NATURE'S WAY PRIMADOPHILUS ORIGINAL ONCE DAILY TRUE POTENCY 5 BILLION CFU TRUE IDENTITY ACIDOPHILUS & RHAMNOSUS TRUE RELEASE TARGETED DELIVERY ENTERIC-COATED FOR ALL AGES
NATURE'S WAY PRIMADOPHILUS REUTERI SUPERIOR PROBIOTIC MULTI STRAIN PLUS SCFOS TRUE POTENCY 5 BILLION CFU ENTERIC-COATED ONCE DAILY
NEW CHAPTER EVERY MAN II WHOLE-FOOD PROBIOTIC MULTI-NUTRIENT, MINERAL & HERBAL COMPLEX
NEW CHAPTER EVERY WOMAN WHOLE-FOOD PROBIOTIC MULTI-VITAMIN, MINERAL & HERBAL COMPLEX
NEW CHAPTER EVERY WOMAN'S ONE DAILY WHOLE-FOOD PROBIOTIC MULTI-VITAMIN, MINERAL & HERBAL COMPLEX
NEW CHAPTER ORGANICS PROBIOTIC NUTRIENTS EVERY MAN II FOR MEN OVER 40 MADE WITH ORGANIC INGREDIENTS
NEW CHAPTER ORGANICS PROBIOTIC NUTRIENTS PERFECT PRENATAL
NEW CHAPTER VITAMIN B COMPLEX WHOLE-FOOD HERBAL PROBIOTIC NUTRIENT COMPLEX
NEW VITALITY RUBY REDS NEW & IMPROVED FORMULA POMEGRANATE, ACAI & MAQUI A DELICIOUS FRUIT AND VEGETABLE WITH POTENT VITAMINS, MINERALS, ENZYMES, HERBS, NUTRIENTS AND PROBIOTICS 100% DAILY VALUE 7 VITA
NOW BERRY DOPHILUS 4 PROBIOTIC STRAINS 2.5 BILLION INPUT NATURAL BERRY FLAVOR XYLITOL SWEETENED CHEWABLES

NSI NUTRACEUTICAL SCIENCES INSTITUTE PROBIOTIC 15-35 15 STRAINS / 35 BILLION MICROORGANISMS PER SERVING
NUTRI-HEALTH FLORA SOURCE MULTI-PROBIOTIC BLEND OF 16 STRAINS / 16 BILLION CELLS CAPSULES
NUTRICOLOGY PROGREENS WITH ADVANCED PROBIOTIC FORMULA
NUTRILITE INTESTIFLORA-7 STICK PACKS
NUTRITION NOW PB8 PRO-BIOTIC ACIDOPHILUS FOR LIFE ORIGINAL FORMULA 14 BILLION GOOD BACTERIA
NUTRITION NOW PROBIOTIC ACIDOPHILUS
NUTRITION NOW RHINO CHEWABLE FOS & ACIDOPHILUS CHEWABLE TABLETS 100% DAILY VALUE OF VITAMIN C
O'DONNELL FORMULAS, INC. FLORA-BALANCE BACILLUS LATEROSPORUS BOD STRAIN CAPSULES
OPTIMUM FREEZE DRIED ACIDOPHILUS LACTOBACILLI
ORIGIN ACIDOPHILUS EASY TO SWALLOW
PB8 PRO-BIOTIC ACIDOPHILUS FOR LIFE
PHILLIPS' COLON HEALTH PROBIOTIC CAPS BAYER
PHILLIPS' COLON HEALTH PROBIOTIC CAPS DAILY PROBIOTIC ONE DAILY BAYER
PREMIER RESEARCH LABS GALLBLADDER-ND PROBIOTIC-DERIVED FORMULA FEATURING ND TECHNOLOGY
PRO-BIOTICS ACIDOPHILUS
PROBIOTIC PEARLS WITH L. ACIDOPHILUS AND B. LONGUM
PROBIOTICA LACTOBACILLUS REUTERI CHEWABLE TABLETS
PROCAPS LABORATORIES FIBERMUCIL U.S.P. PSYLLIUM BULK AND WATER SOLUBLE FIBER FRIENDLY FLORA LACTOBACILLUS
PROJOBA INTERNATIONAL PROBACILLUS PLUS
PURE RESEARCH PRODUCTS, LLC DEL-IMMUNE V LACTOBACILLUS RHAMNOSUS LYSED POWDER
PURITAN'S PRIDE ACIDOPHILUS
PURITAN'S PRIDE POTENT ACIDOPHILUS WITH PECTIN
PURITAN'S PRIDE VITA-FRESH LIFE'S GREENS WITH ADVANCED PROBIOTIC FORMULA 29,205 TOTAL ORAC VALUE VEGETARIAN
RAINBOW LIGHT 50+ MINI-TAB AGE-DEFENSE FORMULA FOOD-BASED MULTIVITAMIN WITH COQ10 1,000 IU VITAMIN D3 PROBIOTICS & DIGES
RAINBOW LIGHT ACTIVE HEALTH TEEN FOOD-BASED MULTIVITAMIN TARGETED TEEN NUTRITION 75 MILLION LIVE ACTIVE PROBIOTICS
RAINBOW LIGHT JUST 1 ONCE NATURALS WOMEN'S ONE MULTIVITAMIN/MINERAL NOW WITH PROBIOTICS
RAINBOW LIGHT KIDS ONE MULTISTARS CHEWABLE MULTIVITAMIN / MINERAL WHOLESOME VEGETABLE CONCENTRATES 15 MILLION ACTIVE PRO
RAINBOW LIGHT MEN'S ONE JUST 1 ONCE FOOD-BASED MULTIVITAMIN ENERGY B-COMPLEX & PROBIOTICS

RAINBOW LIGHT WOMEN'S ONE JUST 1 ONCE FOOD-BASED MULTIVITAMIN 800 IU VITAMIN D3 & PROBIOTICS
RBC DIGESTION FORMULA WITH ENZYMES, PROBIOTICS AND NANOCLUSTERS
RENEW LIFE FLORABEAR FOR KIDS
REPHRESH PRO-B PROBIOTIC FEMININE
REXALL NATURALS GESTAZYME MULTI-ENZYME PROBIOTIC FORMULA ACIDOPHILUS PROBIOTIC FORMULA 1 BILLION ACTIVE CULTURES PER SER
REXALL NATURALS ULTIMATE PROBIOTIC FORMULA ACIDOPHILUS MADE WITH BIO FLORA 2 BILLION ACTIVE CULTURES
RITE AID PHARMACY NATURAL ACIDOPHILUS PROBIOTIC COMPLEX MILK FREE 300 MG
RITE AID PHARMACY PROBIOTIC COLON CARE LACTOBACILLUS ACIDOPHILUS, BIFIDOBACTERIUM LONGUM, B. BIFIDUM PROBIOTIC ONE DAILY
SALUS FLORADIX CALCIUM LIQUID MINERAL NO ADDED SUGAR HERBAL
SAV-ON OSCO CENTRAL-VITE SELECT MULTIVITAMIN/MULTIMINERAL FOR MATURE ADULTS WITH FLORAGLO LUTEIN
SAV-ON OSCO ONE CHOICE THE MOST COMPLETE MULTIVITAMIN WITH HERBS WITH FLORAGLO LUTEIN
SCHIFF DIGESTIVE ADVANTAGE PROBIOTIC GUMMIES
SCHIFF SUSTENEX PROBIOTIC GUMMIES
SHAKLEE BIFIDUS & ACIDOPHILUS OPTIFLORA PROBIOTIC COMPLEX
SHAKLEE OPTIFLORA FOS, INULIN, & MORE PREBIOTIC DIETARY SUPPLEMENT
SHAKLEE OPTIFLORA PREBIOTIC COMPLEX FOS, INULIN, & MORE
SOLARAY MULTIDOPHILUS 12 OVER 20 BILLION MICROORGANISMS 12 PROBIOTIC STRAINS
SOLGAR ADVANCED 40+ ACIDOPHILUS NON-DAIRY LACTOSE FREE
SOLGAR ADVANCED ACIDOPHILUS PLUS 500 MILLION MICROORGANISMS PER USE
SPRING VALLEY 1 PER DAY DOSE ALL NATURAL CRANBERRY WITH VITAMIN C & PROBIOTICS NEW IMPROVED FORMULA
SPRING VALLEY ACIDOPHILUS
SPRING VALLEY PROBIOTIC ACIDOPHILUS 1 BILLION ACTIVE CULTURES
SPRING VALLEY PROBIOTIC MULTI-ENZYME DIGESTIVE FORMULA 1 BILLION ACTIVE CULTURES PER SERVING
SPRING VALLEY PROBIOTIC MULTI-ENZYME DIGESTIVE FORMULA WITH ACTIVE ACIDOPHILUS CULTURES
SPRING VALLEY SUPER STRENGTH PROBIOTIC ACIDOPHILUS 2 BILLION ACTIVE CULTURES
STOCKBRIDGE NATURALS TRI-DOPHILUS
SUNDOWN ACIDOPHILUS XTRA
SUNDOWN NATURALS CHEWABLE ACIDOPHILUS WITH BIFIDUM 1 BILLION ACTIVE CULTURES PER WAFER VEGETARIAN FORMULA
SUNDOWN NATURALS PROBIOTIC ACIDOPHILUS XTRA 40 MILLION LIVE CELLS PER SERVING VEGETARIAN FORMULA

SUNDOWN NATURALS ULTIMATE PROBIOTIC FORMULA ACIDOPHILUS 2 BILLION ACTIVE CULTURES VEGETARIAN FORMULA 1 PER DAY
SUSTENEX DAILY PROBIOTIC 2 BILLION CELLS OF GANEDENBC30
SWANSON ULTRA FLORAGLO LUTEIN 10 MG SOFTGELS
SWANSON ULTRA PROBIOTIC COMPLEX
THE HONEST CO. BABY & TODDLER MULTI POWDER COMPLETE MULTI-VITAMIN + MINERALS + SUPERFOODS + ANTIOXIDANTS + AMINO ACIDS + PROBIOTICS WHOLE-FOOD BASED ULTRA PURE GENTLE & ENRICHING HYPOALLERGENIC 100% N
THE VITAMIN SHOPPE ACIDOPHILUS WITH PECTIN
THE VITAMIN SHOPPE PSYLLIUM HUSK WITH ACIDOPHILUS
THE VITAMIN SHOPPE ULTIMATE '10' PROBIOTIC 13 BILLION ORGANISMS WITH FRUCTO-OLIGOSACCHARIDES ENTERIC COATED
THERALAC PROBIOTIC MASTER 5 HUMAN STRAINS 2 PREBIOTICS 5 + 2 30 BILLION CFU PER CAPSULE WITH LACTOSTIM
TOTAL PROBIOTICS NUTRI-WEST
TRADER JOE'S ACIDOPHILUS & PROBIOTIC COMPLEX 2 BILLION ORGANISMS PER TABLET
TRADER JOE'S ACTIVE 50+ ONCE DAILY MULTIVITAMIN & MINERAL PLUS PROBIOTICS 1000 IU VITAMIN D
TRUBIOTICS DAILY PROBIOTIC FROM THE MAKERS OF ONE A DAY BAYER
TRUNATURE DIGESTIVE PROBIOTIC 10 BILLION ACTIVE CULTURES
TWINLAB ALLERDOPHILUS ACIDOPHILUS CAPSULES HIGH POTENCY 182 MG
ULTIMATE FLORA CRITICAL CARE 50 BILLION EXTRA-STRENGTH PROBIOTIC 10 PROBIOTIC STRAINS RENEW LIFE
ULTIMATE FLORA EXTRA CARE DAILY PROBIOTIC 30 BILLION LIVE CULTURES PER CAPSULE 10 PROBIOTIC STRAINS ONE CAPSULE ONCE A DAY PROBIOTIC RENEW LIFE
ULTIMATE FLORA KIDS PROBIOTIC 3 BILLION LIVE CULTURES PER TABLET PROBIOTIC RENEW LIFE
VIBRANT HEALTH GREEN VIBRANCE 25 BILLION PROBIOTICS PER DOSE FROM 12 STRAINS ORGANIC GREENS & FREEZE DRIED GRASS JUICES
VITAMIN WORLD ACIDOPHILUS & PSYLLIUM HUSK 4 BILLION ACTIVE CULTURES 4,000 MG PSYLLIUM HUSK PER SERVING
VITAMIN WORLD CHEWABLE ACIDOPHILUS WITH BIFIDUS NATURAL STRAWBERRY FLAVOR
WALGREENS ACIDOPHILUS 10 MG
WALGREENS ACIDOPHILUS PLUS CITRUS PECTIN FREEZE DRIED
WINDMILL NATURAL ACIDOPHILUS PRO-BIOTIC BLEND WITH PECTIN
Y.S. ORGANIC BEE FARMS 100% PURE BEE POLLEN CAPSULES MULTI FLORAL, WILD CRAFTED
NEW CHAPTER ORGANICS PROBIOTIC NUTRIENTS CAL MAG BONE HEALTH CALCIUM AND SYNERGISTIC NUTRIENTS IN THEIR MOST ACTIVE FORM
WHOLE FOODS PREMIER FORMULA PROBIOTIC COMPLEX PLUS
NUTRITION NOW PB8 PRO-BIOTIC ACIDOPHILUS FOR LIFE
CVS/PHARMACY ADULT PROBIOTIC 3 BILLION LIVE BACTERIA CELLS PER CAPSULE WITH 8 NATURAL PROBIOTIC STRAINS, INCLUDING 2 BIFIDOBACTERIA STRAINS ONE-A-DAY

CULTURELLE PROBIOTIC NATURAL HEALTH & WELLNESS ONCE DAILY CAPSULES ALL NATURAL PROBIOTIC WITH LACTOBACILLUS GG
RAINBOW LIGHT ACTIVE SENIOR JUST 1 ONCE FOOD-BASED MULTIVITAMIN B-COMPLEX 1,000 IU VITAMIN D3 & PROBIOTICS
NEW CHAPTER ORGANICS PROBIOTIC ALL-FLORA WHOLE FOOD LIVE PROBIOTICS
GARDEN OF LIFE RAW PROBIOTICS ULTIMATE CARE 100 BILLION GUARANTEED! 34 PROBIOTIC STRAINS ONCE DAILY INCLUDES THE CLINICALLY STUDIED REPLENISH BLEND RAW WHOLE FOOD PROBIOTIC
DEFAULT CHILDREN'S PROBIOTIC PACKET
METABIOTIC PROBIOTIC WITH BIO-ACTIVE 12 PROBIOTIC
SPRING VALLEY PROBIOTIC ACIDOPHILUS 2 BILLION ACTIVE CULTURES
DEFAULT GUMMY ADULT PROBIOTIC
NATURE'S SUNSHINE L. ACIDOPHILUS MODIFIED RELEASE PROBIOTIC FORMULA
DAVINCI LABORATORIES OF VERMONT NONDAIRY PROBIOTIC 50 VEGETARIAN / GLUTEN FREE
NATURE MADE DIGESTIVE HEALTH PROBIOTIC 10 BILLION LIVE CELLS ONCE DAILY

Table S2. – Variables included in the adjusted models

	Variables included in all analyses	Variables included only in specific analysis
**Model 1**	Age, sex, ethnicity (Mexican American, other Hispanic, non-Hispanic white), annual family income (<$25000, $25000 to $75000, >$75000), and education (<9th grade, >= 9th grade)	
**Model 2**	Model 1 + alcohol intake, smoking status (never a smoker, current smoker, or former smoker), physical activity (low, intermediate, high), ingested kcal per day, ingested carbohydrates/kcal per day, ingested proteins/kcal per day, ingested fibres/kcal per day, and ingested polyunsaturated/saturated fatty acids ratio.	BMI (all analyses except obesity related analyses);<br>Sodium intake per day (only in hypertension and blood pressure analyses)
**Model 3** **(supplementary)**	Model 1 + alcohol intake, smoking status, physical activity, and DASH dietary pattern score*.<br>* The DASH score is based on 9 target nutrients (sodium, total fat, saturated fat, protein, fiber, cholesterol, calcium, magnesium, and potassium). Individuals meeting the DASH goal were given a score of 1.0 for that nutrient and if they attain a intermediate goal were given a score of 0.5 for that nutrient. The DASH score is the sum of the score for each individual nutrient.	BMI (all analyses except obesity related analyses);<br>Sodium intake per day (only in hypertension and blood pressure analyses)

Table S3. – Odds ratio of disease in subjects exposed to probiotics compared to non-exposed (model 3).

	Model 3, OR (95%CI)	P value
**Obesity**	0.86 (0.78-0.94)	0.002*
**Diabetes**	1.01 (0.84-1.21)	0.937
**Hypertension**	0.79 (0.71-0.88)	<0.001*
**Dyslipidemia**	1.00 (0.89-1.13)	0.957

Model 3: Model 1 + alcohol intake, smoking status, physical activity, and DASH dietary pattern score. Model 3 also includes BMI in all analyses except in the obesity analysis; and includes sodium intake per day only in the hypertension analysis. *statistically significant

Table S4. – Variation of cardiometabolic parameters in participants exposed to probiotics compared to non-exposed (model 3).

	Model 3	P value
BMI, kg/m^2	-0.33 (-0.59 to -0.06)	0.015*
HbA1c [a], %	0.01 (-0.01 to 0.03)	0.416
Glucose [a], mg/dL	-0.07 (-0.82 to 0.68)	0.862
Systolic BP [b], mmHg	-1.54 (-2.38 to -0.70)	<0.001*
Diastolic BP [b], mmHg	-0.89 (-1.49 to -0.29)	0.004*
LDL [c], mg/dL	0.27 (-2.29 to 2.83)	0.836
HDL [c], mg/dL	1.45 (0.71 to 2.18)	<0.001*
Triglycerides [c], mg/dL	-9.62 (-16.42 to -2.82)	0.006*

Model 3: Model 1 + alcohol intake, smoking status, physical activity, and DASH dietary pattern score. Model 3 also includes BMI in all analyses except in the BMI analysis; and includes sodium intake per day only in the BP analyses.

[a] Excluding participants treated with anti-hypertensive drugs. [b] Excluding participants treated with anti-dyslipidemic drugs. [c] Excluding participants treated with antidiabetic drugs. *statistically significant
